# Next-Generation Sequencing Identifies Potential Actionable Targets in Paediatric Sarcomas

**DOI:** 10.3390/jpm11040268

**Published:** 2021-04-03

**Authors:** Antonio Juan Ribelles, Pablo Gargallo, Pablo Berlanga, Vanessa Segura, Yania Yáñez, Bárbara Juan, Marta Salom, Margarita Llavador, Jaime Font de Mora, Victoria Castel, Adela Cañete

**Affiliations:** 1 Paediatric Oncology and Hematology Unit, Hospital U I P La Fe, Av. Fernando Abril Martorell, 106, 46026 Valencia, Spain; canyete_ade@gva.es; 2 Clinical and Translational Oncology Research Group, Instituto de Investigación Sanitaria La Fe, 46026 Valencia, Spain; gargallo_pabtat@gva.es (P.G.); segura_van@gva.es (V.S.); yanyez_yan@gva.es (Y.Y.); jaime.fontdemora@gmail.com (J.F.d.M.); castel_vic@gva.es (V.C.); 3 Department of Child and Adolescent Cancer, Institute Gustave Roussy, 114 Rue Edouard Vaillant, 94805 Villejuif, France; berlanga_pab@gva.es; 4 Facultad de Medicina, Universidad de Valencia, Av. Blasco Ibáñez 15, 46010 Valencia, Spain; babs_jr96@hotmail.com; 5 Paediatric Orthopedic Surgery, Hospital U i P La Fe, 46026 Valencia, Spain; msalomta@yahoo.es; 6 Pathology Department, Hospital U i P La Fe, 46026 Valencia, Spain; llavador_mar@gva.es

**Keywords:** paediatric sarcomas, next-generation sequencing, precision medicine, clinical translation, targeted drugs

## Abstract

**Background:** Bone and soft-tissue sarcomas represent 13% of all paediatric malignancies. International contributions to introduce next-generation sequencing (NGS) approaches into clinical application are currently developing. We present the results from the Precision Medicine program for children with sarcomas at a reference centre. **Results:** Samples of 70 paediatric sarcomas were processed for histopathological analysis, reverse transcriptase polymerase chain reaction (RT-PCR) and next-generation sequencing (NGS) with a consensus gene panel. Pathogenic alterations were reported and, if existing, targeted recommendations were translated to the clinic. Seventy paediatric patients with sarcomas from 10 centres were studied. Median age was 11.5 years (range 1–18). Twenty-two (31%) had at least one pathogenic alteration by NGS. Thirty pathogenic mutations in 18 different genes were detected amongst the 22 patients. The most frequent alterations were found in *TP53*, followed by *FGFR4 and CTNNB1*. Combining all biological studies, 18 actionable variants were detected and six patients received targeted treatment observing a disease control rate of 78%. Extrapolating the results to the whole cohort, 23% of the patients would obtain clinical benefit from this approach. **Conclusions:** Paediatric sarcomas have a different genomic landscape when compared to adult cohorts. Incorporating NGS targets into paediatric sarcomas’ therapy is feasible and allows personalized treatments with clinical benefit in the relapse setting.

## 1. Introduction

Paediatric sarcomas account for over 20% of all paediatric solid malignant cancers and represent 13% of all paediatric malignancies [1]. They also contribute substantially to cancer-related mortality and morbidity. With more than 70 histologic subtypes, sarcomas can arise from a primitive mesenchymal cell from almost every tissue in the human body and are classified into two main groups: soft tissue sarcomas (STS) and bone sarcomas (BS). The highest incidence rates in children are reported amongst rhabdomyosarcoma (RMS), osteosarcoma and Ewing’s sarcoma (EWS). Although each subtype has a different phenotype and genetic profile, they are classified into two molecular groups: a *genetically complex* group with a high mutational burden and complex karyotype, and a *genetically simple* group containing a single and disease-specific translocation, amplification or mutation with a silent genomic background [2]. Most paediatric sarcomas are included in the second group as they are mostly characterized by chromosomal translocations that result in hybrid genes acting as drivers that are critical for sarcoma-genesis [3].

Paediatric RMS protocols currently classify this tumour based on the presence of *PAX/FOXO1* translocation and distinguish between fusion positive or fusion negative RMS [4]. The genetic profile of EWS is dominated by the driving reciprocal chimeric translocation between *EWSR1* and a variety of *ETS* partner transcription factors [5]. These gene fusions act as oncogenic transcription factors that trigger transcriptomic and epigenetic dysregulations that explain the tumour’s biology [6,7]. In contrast to EWS and RMS, osteosarcoma shows an extremely complex and unstable genome but without a remarkable repetitive pattern [8]. In most clinical settings, sarcomas involving translocations are detected by fluorescence in situ hybridization (FISH) and reverse transcriptase polymerase chain reaction (RT-PCR). Translocations are used by clinicians mostly as diagnostic markers [9]. However, the resulting chimeric proteins of these translocations are not easily druggable and hinder the development of inhibitors. Table 1 shows the most frequent fusion transcripts in paediatric sarcomas.

Both STS and BS display a highly aggressive behaviour. During recent decades, addition of systemic chemotherapy has improved outcome of localized tumours resulting in the survival of two-thirds of patients. However, metastatic and relapsed sarcomas still have very poor survival rates. Despite the knowledge gained in cancer biology, aetiology and in the implementation of novel diagnostic techniques and omics, scarce improvement has been observed in advanced stage STS and BS.

During the last years, precision and quality criteria for the diagnosis of paediatric cancers, including sarcomas, has experienced an increased demand. New techniques have been introduced that complement pathological diagnosis including immunochemistry, FISH, RT-PCR and next-generation sequencing (NGS). These demands have been gradually assumed by clinicians, pathologists, geneticists and molecular biologists in tertiary reference hospitals. In addition, precision medicine programs have been developed in order to expand our knowledge of tumour biology and defeat cancer with more precise pharmacological targets [10,11,12,13]. We present the results for paediatric sarcomas from the Precision Medicine program for children and adolescents with solid tumours in relapse/progression carried out at a national reference centre for paediatric sarcomas. This program has received samples from collaborative centres, providing a national perspective [14]. Since September 2019, these studies are routinely carried out at diagnosis in every paediatric sarcoma.

## 2. Materials and Methods

### 2.1. Study Subjects

A total of 70 sarcoma samples from paediatric patients treated at a reference institution for paediatric sarcomas or at other Spanish centre from February 2015 to March 2020 were included. Thirty patients were analysed at diagnosis and forty patients were studied at relapse or refractory disease.

The program was approved by the Ethics Committee of the centre. Parents signed the informed consent and were informed about the possibility of finding germline mutations and accepting or refusing to be informed. Consent was also required when performing NGS studies at diagnosis. Every procedure was performed according to the Declaration of Helsinki.

### 2.2. Study Samples

Fresh tumour samples were requested. Paraffined-embedded tumours and/or pre-treatment biopsies were only used if fresh samples were unavailable. Peripheral blood samples were simultaneously collected in 45 cases. All tumour samples were reviewed by a board-certified pathologist to confirm histology and estimate tumour cell content. Immunochemistry techniques (p-AKT, PDL1, p-EGFR, c-KIT, PTEN, Her2neu, p53) and FISH (NTRK1/3, ALK, BRAF) were also performed. Only samples with >30% tumour cell content were considered for further genomic testing; the rest were excluded from the study. The selected tumour material and peripheral blood samples were sent to a biobank for DNA extraction and subsequently to the laboratory for sequencing analysis. In some cases, based on previous literature and according to the sequencing results obtained for each tumour type, studies were completed with SNP array analysis.

### 2.3. DNA Extraction

DNA extraction from tumour and blood samples was carried out using the QIAamp^®^ DNA Investigator kit (QIAGEN^®^ ref. 56504) or QIAamp^®^ DNA Mini Kit (QIAGEN^®^ ref. 51), 304, respectively, following manufacturer instructions. The concentration and absorbance ratios were measured with NanoDrop 2000^®^13.

### 2.4. RNA Extraction and cDNA Generation

RNA was extracted with the RecoverAll™ Total Nucleic Acid Isolation Kit following manufacturer’s protocol. Total RNA was quantified with the with the Qubit™ RNA HS Assay Kit (ThermoFIsher Scientific), and cDNA was obtained with the SuperScript™ IV VILO™ Master Mix.

### 2.5. Sequencing Studies, Data Interpretation and Variant Calling

Commercial and customized NGS panels that included the consensus gene list and that produced an average coverage of 1000X and homogeneity with a minimum of 85% were used for the analysis of relapse or refractory patients: Ion Ampliseq Cancer Hotspot Panel v2 (Thermo Fisher Scientific), Human Comprehensive^®^ Cancer Panel (Qiagen©), Paediatric-OncoPanelDx^®^ (Imegen) and Onconano Gene Panel (Paediatric Oncology Group-IIsLaFe). For analysis of newly diagnosed samples, the Oncomine Childhood Research Assay^®^ was used (Ref: A36485).

Gene panels included at least the following: *ABL1, AKT1, ALK, BRAF, CDKN2A, CSF1R, CTNNB1, EGFR, ERBB2, ERBB4, EZH2, FGFR1, FGFR2, FGFR3, FLT3, GNA11, GNAQ, HRAS, IDH1, IDH2, JAK2, JAK3, KIT, KRAS, MET, MPL, NRAS, PDGRFA, PIK3CA, PTEN, PTPN11, RB1, RET, SMARCB1, SMO,* and *TP53*. Mutations in other genes were not evaluated in this study.

For NGS data analysis, variant calling was based on the genome version GRCh37 (hg19). Genetic variants detected in both, blood and paired tumour samples were classified as germline variants, whereas variants detected exclusively in tumours were categorized as somatic variants.

Variant annotation was carried out applying an algorithm of filters in order to discard non-clinically relevant variants: those with an allelic frequency < 5%, changes in non-coding regions (excluding those variants in exon splicing sites +/− 10 nucleotides), synonymous variants (excluding those coding variants nearby splicing sites +/− 4 positions), variants with high frequency in the general population (MAF > 0.01), and polymorphic changes (SNPs) without clinical relevance found in healthy population or described as benign by several sources or our genomic database. The remaining variants were classified according to international recommendations as pathogenic, likely pathogenic, benign, likely benign or of uncertain significance [13] based on literature and specific disease databases (ClinVar, COSMIC, HGMD, St Jude PeCan or CiVIC).

Pathogenic or likely pathogenic variants were reviewed and approved by the Paediatric molecular tumour board (PMTB) committee, and further confirmed using direct Sanger sequencing. Actionable variant was referred as a genomic change that suggests an alteration to biological activity that could be targeted with a specific therapy already used in vivo. Targeted therapies were preferentially recommended to be administered within clinical trials but also as compassionate use basis if trials were not available. Median time between biopsy/surgery and molecular tumour board recommendation was 5 weeks.

### 2.6. Paediatric Molecular Tumour Board Discussion

The PMTB was created in November 2014 and composed of paediatric oncologists, pharmacologists, geneticists, pathologists, molecular biologists and bioinformatics. The PMTB established the consensus gene panel for the NGS analysis. After the completion of pathological and genomic studies, results were discussed in periodical meetings in the PMTB and a final report was transferred to the corresponding physician. The workflow was based on previous pilot studies [14,15]. Clinicians were then responsible for deciding if the targeted treatment was indicated at that moment and if the recommendation could be implemented as standard of care, included as off-label treatment or within a clinical trial (usually early phase). The benefit from the recommended treatments was eventually evaluated by clinical and radiological findings. In the patients that were treated in clinical trials RECIST 1.1 criteria were used.

## 3. Results

### 3.1. Clinical Characteristics

**Figure 1:**
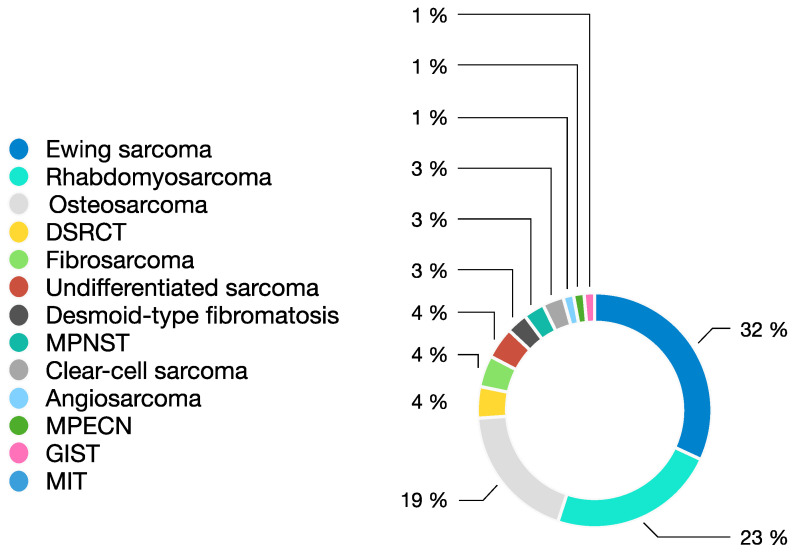
Distribution of sarcoma type amongst reported cases. DSRCT: desmoplastic small round cell tumour; MPNST: malignant peripheral nerve sheath tumour; MPECN: malignant perivascular epithelioid cell neoplasm; GIST: gastrointestinal stromal tumour; MIT: myo-fibroblastic inflammatory tumour.

A total of 70 paediatric and adolescent patients with STS and BS from 10 Spanish cooperating sites were included in a 5-year period from February 2015 to March 2020. Patients’ median age at study entry was 11.5 years with a range of 1–18 years (6 patients 0–4 years, 16 patients 5–8 years, 20 patients 9–12 years and 28 patients 13–18 years old). Forty-one per cent of the patients were female (29) and 59% were male (41). Distribution of tumour type is shown in Figure 1. The most frequent tumours were EWS (*n* = 22), RMS (*n* = 16) and osteosarcoma (*n* = 13). Thirty patients were studied at diagnosis (43%), 22 patients at first relapse (31%), 15 patients at second or successive recurrences (21%) and three patients when found to be refractory to first line treatment (4%). Somatic variants described as pathogenic or likely pathogenic using international system classifications [13] were reported.

### 3.2. NGS Results

Twenty-two out of 70 patients (31%) had at least one pathogenic or likely pathogenic alteration identified by NGS as with a mean of 1.4 mutations per patient. Most of the cases had one unique mutation. A total of 30 different pathogenic or likely pathogenic mutations in 18 different genes were detected amongst the 22 patients. Mutations were detected in relapsed or refractory sarcomas (57%) and also at first diagnosis (43%).

**Figure 2:**
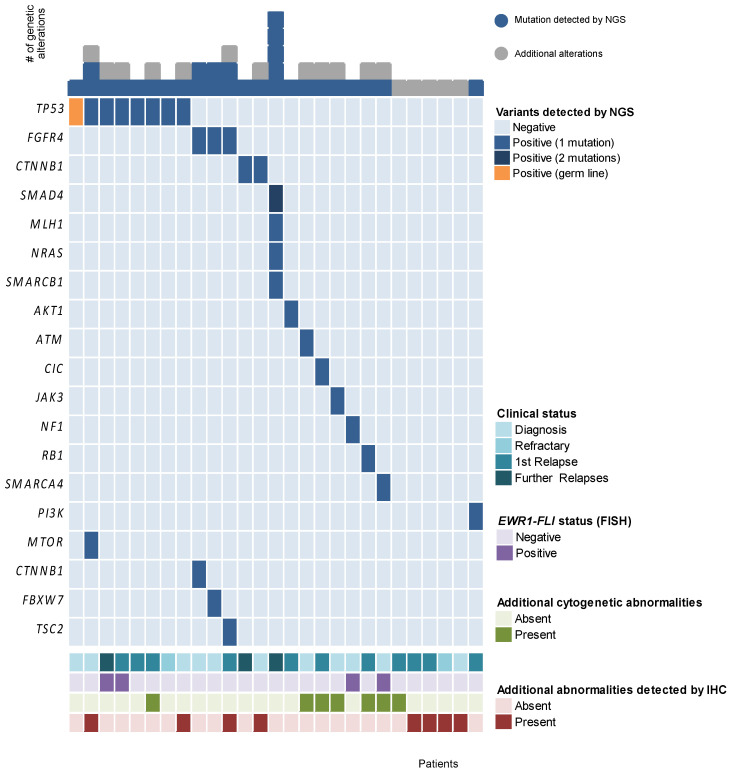
Molecular alterations observed in the 27 patients (each column represents one patient). IHC: immunochemistry.

Diagnostic sarcoma fusion genes detected by FISH or RT-PCR were only used for diagnosis but were not considered for precision medicine recommendations as no targeted treatments are available for these alterations to date. Overall, *TP53* was the most frequently affected gene (27%) and preferentially identified in EWS, RMS and angiosarcoma. Three embryonal rhabdomyosarcomas harboured alterations in *FGFR4* whilst the two aggressive fibromatosis and an embryonal RMS had *CTNNB1* mutations. Identified gene and variant alterations are shown in Table 2. Including information obtained by complementary techniques (immunochemistry and FISH) up to 27 patients had an identified alteration (39% of the cases). A summary of the molecular alterations spotted in these 27 patients is shown in Figure 2.

### 3.3. Clinical Translation

**Figure 3:**
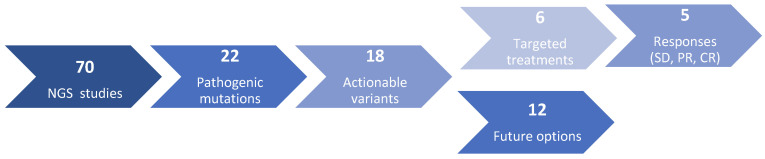
Workflow diagram showing the next-generation sequencing (NGS) process and targeted treatments drawn from the study.

After discussion of the biological results in the PMTB, 18 actionable variants (26%) were identified and formal recommendations were submitted to the respective physicians. RMS was the tumour in which more actionable variants were observed (39%), particularly embryonal histology (28%). Two osteosarcoma patients presented actionable alterations. Despite the number of EWS cases included in the study (22), only one patient had an actionable variant. This result points out the difficulties in implementing a precision medicine strategy in EWS tumours. A workflow diagram detailing the process can be observed in Figure 3.

Six patients out of the whole cohort received targeted treatment (9%), observing clinical benefit in five of them (78%):

A thirteen-year-old female with a radio-induced abdominal malignant nerve sheath tumour, 10 years after neuroblastoma treatment, was found to have a mutation in *ATM.* After radical surgery and standard chemotherapy, she underwent disease progression. Targeted treatment with poly ADP-ribose polymerase (PARP) inhibitor Olaparib and temozolomide was administered, resulting in disease stabilization during one month with clear disease control. She received treatment during two months before a subsequent progression.

A twelve-year-old female with malignant perivascular epithelioid cell kidney tumour with lung metastasis was found to have positive p-AKT with immunochemistry and targeted treatment with sirolimus and sorafenib was initiated after observing no response to classic sarcoma chemotherapy. A slight response was observed in tumour size and the disease was stabilised according to RECIST 1.1 criteria for a five-month period. This achievement had not been possible with the previous schedules administered. Thirdly, a nine-year old female with first local and metastasic osteosarcoma relapse with positive mTOR immunochemistry was treated with an oral mTOR inhibitor during a two-month period after failure of standard treatments. Unfortunately, progression was observed after the third month.

Another twelve-year-old male was affected by a mediastinal myo-fibroblastic inflammatory tumour with ALK translocation detected by FISH. Disease progression was observed after standard chemotherapy (IVA regime) and surgery. Targeted treatment with *ALK* inhibitor ceritinib was started and a very good partial response was observed. Finally, a thirteen-year-old female with a stomach GIST with positive c-KIT diagnosis (CD-117) by immunohistochemistry is currently receiving imatinib after radical surgery and has achieved complete response.

Future treatment options were available for 12 patients (17%) that are at the moment in complete response or receiving other standard treatments. However, in these cases it is important to consider that the mutational profile of the relapsed tumour may be different from the primary tumour at diagnosis Altogether, implementing NGS with complementary diagnostic techniques such as immunohistochemistry and FISH in a precision medicine approach for targeted treatment of sarcomas, a disease control rate of 23% would potentially be achieved. The summary of the recommendations and clinical responses are shown in Table 3.

## 4. Discussion

Genetic variation is one of the main characteristics of paediatric sarcomas. This is mostly explained because despite being originated from a mesenchymal cell, they constitute different histologic entities with different genomic landscapes that explain their unequal behaviours. Beside pathology, chromosomal segmental aberrations, [16] changes in ploidy and specific gene alterations are routinely used in order to guide intensity of treatment in paediatric oncology protocols.

It is worth noting important differences spotted when comparing adult with paediatric NGS studies in sarcomas. [17] Epidemiologically, sarcomas represent less than 1% of all solid malignant cancers in the adult population while they represent 20% of all Paediatric solid malignant cancers. Therefore, the first main difference lies in the fact that the magnitude of the problem is proportionally much higher in the paediatric population. Furthermore, adult type cancers such as epithelial neoplasms arise after accumulation of multiple sequential mutations directly linked to environmental exposures, and arise within differentiated adult tissues [18,19]. Mesenchymal tumours such as sarcomas appear both in adult and paediatric population. However, specific histologic subtypes and clinical progression are age-dependent, suggesting differential pathogenetics and underlying molecular mechanisms for tumour initiation and clinical behaviour in the different age subgroups [18].

In this study, we found that the overall mutational load in our cohort was relatively low when compared to adult studies [20]. In the adult cohort studied by Groisberg et al. [21], 95 out of 102 patients (93%) had at least one genomic alteration identified with a mean of six mutations per patient while in our paediatric cohort only 31% of the patients harboured a genomic alteration with a mean of 1.4 mutations per patient. The magnitude of this difference is so overwhelming that makes the point relevant despite possible differences between the panels used. This might be explained by the fact that adult sarcomas are mostly driven by mutagenic exposure from environmental factors, whereas most paediatric cancers contain a relatively small number of mutations [20] and frequently display unique gene rearrangements. Although this restricts the targeted treatment to available drugs, it also makes them attractive candidates for drug discovery [15]. On the other hand, a precision medicine approach in paediatric sarcomas has several limitations because many of the subtypes contain a single disease-specific driver mutation whose oncogenic effect predominates over other possible passenger mutations detected by NGS.

In order to improve outcome, international efforts amongst cooperative groups have been carried out developing genomic precision medicine programs. These programs aim to bring NGS approaches into the clinical practice and require the identification of patients that might benefit from targeted therapies. Once these targets are identified, in the paediatric population it is important to communicate these results, as well as possible toxicities observed from a compassionate use basis, as dosing is more complex when compared to the adult population. Hence, the importance of promoting paediatric phase I clinical trials in order to titrate infant dosing.

In this study, we conclude that the most frequent somatic mutation observed in paediatric sarcomas occurs in *TP53* (27% of the pathogenic mutations detected by NGS). This information correlates with adult sarcoma cohorts [21]. Xiaosheng et al. [22] compared overall survival (OS) time between *TP53-*mutated and *TP53*-wildtype cancers in 20 adult cancer types. They reported that patients with *TP53* mutations had lower survival compared with those without *TP53* mutations in colon, lung and pancreas adenocarcinoma, acute myeloid leukaemia and other epithelial cancers. In paediatric oncology, the clinical significance of somatic *TP53* mutations remains unrecognized and no routine testing or therapy intensification is considered. Recent studies suggest that mutation in *TP53* in localized EWS is not a reliable prognostic marker [23]. In order to target *TP53*, small molecules that reactivate mutant p53 by restoring wild-type conformation have been identified by various approaches. *APR-246* alone is currently being tested in prostate or ovarian cancers or in combination with azacytidine in myeloid malignancies in adult phase I-II trials. No studies are currently recruiting a paediatric population.

Mutations in Fibroblast Growth Factor Receptor 4 (*FGFR4*) have also been described in paediatric sarcomas, most outstandingly in RMS. Higher *FGFR4* expression in RMS has been associated with advanced-stage cancer and poor survival [24]. *FGFR4* pathogenic mutations appear in 33% of the embryonal RMS studied in our cohort and all of them received a targeted recommendation therapy. *FGFR4* codifies for a cell surface tyrosine kinase (TK) receptor that is involved in normal myogenesis and muscle regeneration. It has been reported that human embryonal RMS cells have increased FGFR4 mRNA expression compared to normal human myoblasts, and FGFR4 pathway blockade decreases proliferation [25]. In fact, over-expression and mutational activation of *FGFR4* has been reported in RMS, promoting tumour progression. *FGFR4* signalling is also a common mechanism of oncogenesis in fusion positive RMS (usually alveolar subtype) [25].

Alterations in *FGFR4* are clinically relevant because they are actionable targets in patients with RMS. A new generation of multikinase inhibitors is under current development such as ponatinib (AP-24534), an orally administered TK inhibitor that was initially developed as an inhibitor for *BCR-ABL*. Ponatinib recently received FDA approval for the treatment of adult patients with Philadelphia chromosome positive acute lymphoblastic leukaemia and chronic myeloid leukaemia resistant to other TK inhibitors. Inhibition profile of ponatinib includes other TKs such as c-KIT, PDGFR, FLT3, SRC and FGFR [26]. Moreover, inhibition of FGFR family members with ponatinib has been demonstrated in preclinical models with bladder cancer, endometrial cancer, breast, lung and colon cancer. Samuel Q. Li et al. [26] tested a panel of RMS cell lines over-expressing FGFR4, all of them exhibiting sensitivity to five different TK inhibitors including ponatinib, cediranib, nintedanib, dovitinib and danusertib. They observed that ponatinib resulted in being the most powerful FGFR4 inhibitor, inhibiting both mutated and wild-type FGFR4 cell growth. It also inhibited tumour development expressing FGFR4 in vivo [26]. Currently, ponatinib is being tested in clinical trials including for paediatric patients (NCT03934372) [25,27]. Erdafitinib is also being tested in a phase II trial for tumours with FGFR mutations. (NCT03210714).

The *CTNNB1* gene provides instructions to form the protein beta-catenin. The relationship between the Wnt/beta-catenin signalling pathway and desmoid-type fibromatosis (DTF) has been widely studied and it has been reported that the vast majority of DTF tumours (up to 85%) harbour a mutation in exon 3 of the *CTNNB1* gene (beta-catenin) [28]. These mutations lead to an abnormally stable beta-catenin protein that is more resistant to proteolytic degradation and accumulates within the cells. Excess of beta-catenin promotes an uncontrolled proliferation of cells, allowing the formation of DTF [29].

Therapeutic options targeting the Wnt/beta-catenin signalling pathway are limited and have not been tested in paediatric population. Accumulation of beta-catenin in the nucleus triggers transcription of Wnt-specific genes responsible for the control of cell fate decisions. The development of drugs targeting mutated or altered beta-catenin signalling, or its interaction with CBP, TCF, GSK3β or APC (which are essential to complete its function) has been difficult due to the toxicity of the new compounds. Several of them are currently in Phase 1 clinical trials, such as the PRI-724 molecule (NCT01302405, NCT02413853, NCT01764477, and NCT01606579) that prevents the interaction of beta-catenin with CBP. Despite these and other approaches, there are no clinical trials available for paediatric patients with Wnt/beta-catenin inhibitors [30]. All DTF studied in our cohort harboured mutations in *CTNNB1*.

In the study, a patient with malignant nerve sheath tumour and *ATM* mutation was treated with PARP inhibitors in combination with temozolomide. The ataxia telangiectasia gene (*ATM*), localized in 11q22-q23, plays an important role in maintaining genomic integrity. It regulates the double-strand DNA breaks repair and activates different checkpoints in the cell cycle. *ATM* is associated with some types of leukemia and lymphoma and it has also been described in neuroblastoma with 11q deletion. Poly ADP-ribose polymerase (PARP) is a protein that signals DNA damage and contributes towards DNA repair [31]. PARP catalyses the addition of ADP-ribose to DNA, helicases, topoisomerases and histones. It also has a critical role in transcription, cellular replication, gene regulation, differentiation, spindle maintenance and protein degradation. PARP inhibition produces persistent single strand DNA breaks leading to double strand DNA breaks and finally produces DNA damage leading to apoptosis and cell cycle arrest. Preclinical studies show that *ATM* mutated neuroblastoma cells also succumb to apoptosis when treated with PARP inhibitors and neuroblastomas with 11q deletion are extremely sensitive to conventional chemotherapy combined with PARP inhibitors. The patient in the study managed a short period of stable disease but progressed rapidly afterwards [31]. Other mutations considered as uncertainly significant in *ATM* have been detected but no recommendations were issued because no previous clinical evidence was found. Currently, early phase trials with PARP inhibitors are recruiting paediatric patients with diverse malignancies.

Recent studies in RMS have revealed recurrent mutations in the *RAS* pathway, particularly affecting NRAS. Dolghik et al. [32] demonstrated that *PIK3CA* played a critical role in the activation of the PI3K/AKT/mTOR pathway in *NRAS* mutant RMS. They noted that NRAS-mutated RMS cells particularly relied on *PIK3CA* to prevent cell death upon *NRAS* silencing or *MEK* inhibition. Their data showed that specific *PIK3CA* knockdown was sufficient to cooperatively trigger cell death together with pharmacological *MEK* inhibition. In addition, pharmacological inhibitors of *MEK* or *NRAS* knockdown synergize with the *PIK3CA* specific inhibitor BYL719 to trigger cell death in *NRAS*-mutated RMS cells. All this data supports the rationale for the combination of *MEK* and *PIK3CA* specific inhibitors in *NRAS* mutated RMS. This recommendation is a future option for one of the patients studied in our cohort.

In this study, a patient diagnosed with c*-KIT* positive (CD-117) GIST was treated with imatinib and so far, has maintained complete response after surgery. Another patient with *ALK*+ myo-fibroblastic inflammatory tumour received treatment with ceritinib obtaining a partial response. Both of these rare sarcomas have a classical alteration that has been widely reported before.

In conclusion, we have observed that the incorporation of NGS results together with ancillary studies into paediatric sarcoma clinical practice is feasible and allows personalized treatments with acceptable disease control rates in the relapse setting. At the moment, as the integration NGS as a routine diagnostic technique has been limited, this is difficult to estimate, although the situation is changing and sequencing studies are gradually becoming widespread [33,34,35]. Further investigations are required to confirm this hypothesis.

In this study, up to 23% of patients would obtain clinical benefit by implementing this precision medicine approach complementing routine diagnostic techniques. However, it is worth noting that many of the NGS results are at the moment possible future options for patients receiving standard therapies and that the response rate in these cases is still awaiting proper evaluation. Another fact is that some of the recommendations were based on IHC and this highlights the importance of routine diagnostic studies and probably the need to explore in larger cohorts the usefulness of NGS in paediatric sarcomas. Finally, it is also important to further assess the cost-effectiveness of NGS in paediatric sarcomas and whether incorporating NGS to standard of care really justifies the costs of the exercise.

Although the understanding of paediatric sarcomas’ biology has improved in a relatively short period of time, outcomes in high-risk tumours remain poor and regarding new therapeutic strategies, very few advances have been remarkable. This emphasizes that strong, international efforts are still required in order to improve implementation of new diagnostic techniques, accelerate paediatric drug development and access to clinical trials in childhood. Finally, we would like to stress the importance of treating childhood, adolescent and young adult sarcomas and other types of cancers in specialized units, with all the available expertise and distinct requirements involving this particular population.

## Figures and Tables

**Table 1:** Main fusion transcripts in paediatric sarcomas: chromosomal translocation, gene transcripts and expected frequencies. RMS: rhabdomyosarcoma; STS: soft-tissue sarcoma.

Diagnosis	Translocation	Fusion	Frequency
Alveolar rhabdomyosarcoma	t(2;13)(q35;q14) t(1;13)(p36;q14)	PAX3/FOXO1	60%
		PAX7/FOXO1	20%
Ewing Sarcoma	t(11;22)(q24;q12)	EWSR1/FLI1	85%
	t(21;22)(q22;q12)	EWSR1/ERG	10%
	t(7;22)(p22;q12)	EWSR1/ETV1	<1%
	t(2;22)(q35;q12)	EWSR1/FEV	<1%
	t(16;21)(p11;q22)	FUS/ERG	<1%
Desmoplastic small round cell tumor	t(11;22)(p13;q12.2)	EWRS1/WT1	>95 %
Infantile fibrosarcoma	t(12;15)(p13.2;q25.3)	ETV6/NTRK3	70 %
Synovial sarcoma	t(X;18)(p11.2;q11.2-11.23)	SS18/SSX1	64%
	t(X;18)(p11.2;q11.2-11.23)	SS18/SSX2	35%
	t(X;18)(p11.2;q11.2-11.23)	SS18/SSX4	1%
Clear cell soft tissue sarcoma	t(12;22)(q13;q12)	EWSR1/ATF1	>90%

**Table 2:** Tumour sequencing results: Mutated gene, variant, sarcoma subtype and clinical status. DSRCT: desmoplastic small round cell tumour; RMS: rhabdomyosarcoma; EWS: Ewing sarcoma; DTF: desmoid-type fibromatosis; MPNST: malignant peripheral nerve sheath tumour.

Gene	Varint	Tumour Type	Clinical Status
*TP53*	c.1040C > T (p. A347V) c.559G > C (p. G187R) c.906_907delCCins TT (p. R303 ) c.742C > T (p. R248W) c.404G > T (p.C135F) c.817C > T (p.R272C) c.448A > G (p. Y163C) c.614A > G (p. Y205C)	DSRCT Embryonal RMS Angiosarcoma EWS EWS Alveolar RMS EWS EWS	Relapse Diagnosis Diagnosis Diagnosis Relapse Refractory Relapse Relapse
*FGFR4*	c.1648G > A (p. V550M) c.1648 G > C (p. V550L) c.1648 G > C (p. V550L)	Embryonal RMS Embryonal RMS Embryonal RMS	RelapseDiagnosis Diagnosis Diagnosis
*CTNNB1*	c.134C > T (p. S45F) c.134C > T (p. S45F) c.133_134delTCinsCT (p. S45L)	DTF DTF Embryonal RMS	Diagnosis Relapse Diagnosis
*SMAD4*	c.302G > A (p. W101 ) c-370G > A (p D124N)	Alveolar RMS Alveolar RMS	Relapse Relapse
*ATM*	c.7032G > A (p W2344 )	MPNST	Diagnosis (secondary tumour)
*NRAS*	c.176C > T (p A59V)	Alveolar RMS	Relapse
*CIC*	c5939_5943del (p G1980Vfs 78)	Clear cell renal sarcoma	Relapse
*FBXW7*	c.1394G > A (p R465H)	Embryonal RMS	Diagnosis
*RB1*	c. 361 C > T (p.Q121 )	Osteosarcoma	Relapse
*AKT1*	c.138C>A (p D46E)	MPNST	Relapse
*JAK3*	c.2164G > A (p V722I)	EWS	Diagnosis
*PI3K*	c.1624G > A (p E542K)	Embryonal RMS	Relapse
*SMARCB1*	c.1135 > A (p A379T)	Alveolar RMS	Relapse
*MLH1*	c.1138G > A (p A380T)	Alveolar RMS	Relapse
*MTOR*	c.6644C > T (p S2215F)	Angiosarcoma	Diagnosis
*TSC2*	c.5158C > T (*p* R1720W)	Embryonal RMS	Relapse
*SMARCA4*	c.1135 > A (*p* A379T)	DSRCT	Diagnosis
*NF1*	c.2087G > A (*p* W696 )	EWS	Diagnosis

**Table 3:** Clinical translation: Actionable variants detected, molecular tumour board recommendations, targeted treatments administered and results observed. EWS: Ewing sarcoma; IQ: immunochemistry; RMS: rhabdomyosarcoma; DTF: desmoid-type fibromatosis; MPNST: malignant peripheral nerve sheath tumour; LOH: loss of heterozygosity; CNV: copy number variation; MIT: myo-fibroblastic inflammatory tumour; GIST: gastrointestinal stromal tumour; MPECN: malignant perivascular epithelioid cell neoplasm; PARP: poly ADP-ribose polymerase.

	Actionable Variant	Other Biologic Information	Committee Recomendation	Treatment Administered	Maximum Response
EWS	*TP53* c.742C > T (p. R248W)	*PDL-1* + (IQ 5%)	*PRIMA-1/PD-L1* inhibitors	No (future option)	−
Embryonal RMS	*FGFR4*c.1648 G > C (p. V550L) *FBXW7* c.1394G > A (p. R465H)	−	Ponatinib/Erdafitinib	No (future option)	−
Embryonal RMS	*TP53* c.559G > C (p. G187R) in germline	*P-AKT* + (IQ 50%)	*mTOR* inhibitor. Li-Fraumeni follow-up	No (future option)	−
Embryonal RMS	*TSC2* c.5158C > T (p. R1720W) *FGFR4* c.1648G > A (p. V550M)	*mTOR* + (IQ 100% cytoplasm)	*mTOR* inhibitor	No (future option)	−
Embryonal RMS	*PI3K* c.1624G > A (p. E542K)	−	*mTOR* inhibitor	No (future option)	−
Embryonal RMS	*FGFR4* c.1648 G > C (p. V550L) *CTNNB1* c.133_134delTCinsCT (p. S45L)	−	Ponatinib/Erdafitinib	No (future option)	−
Alveolar RMS	*SMAD4* c.302G > A(p. W101 ) *SMAD4* c-370G > A (p. D124N) NRAS c.176C > T (p. A59V)	−	Palbociclib + Venetoclax	Yes (<1 month)	PD
Alveolar RMS	*TP53* c.817C > T (p. R273C)	−	*PRIMA-1*	No (future option)	−
Angiosarcoma	*TP53c.906_907delCCinsTT (p. R303 ) MTOR* c.6644C > T (p. S2215F)	*P-AKT* + (IQ 60% membrane and cytoplasm) Patient with Xerodermapigmentosum	*mTOR* inhibitor	No (future option)	−
DTF	*CTNNB1* c.134C > T (p. S45F)	IQ betacatenin +	Beta-catenin inhibitor	No (future option)	−
DTF	*CTNNB1* c.134C > T (p. S45F)	IQ betacatenin +	Beta-catenin inhibitor	No (future option)	−
MPNST	*ATM* c.7032G > A(p. W2344 )	11q deletion	*PARP* inhibitor	Yes (2 months)	SD
Osteosarcoma	*RB1* c. 361 C > T (p. Q121 )	Gain chromosomes: +14, + 20, + 21, Segmental imbalances: 2p, 17q. LOH 3, 16. *PD-L1* + (IQ 20%)	*PD-L1* inhibitors	No (medical decision)	−
Osteosarcoma	−	*mTOR* + (IQ 60% cytoplasm)	*mTOR* inhibitor	Yes (2 months)	SD
Undifferentiated sarcoma	−	CNV: Deletion in genes *ARID1A, MTOR, NRAS, SDHB*	Tazemetostat/Vorinostat	No (future option)	−
MIT	−	FISH: *ALK* +	Ceritinib	Yes (3 months)	PR
GIST	−	IQ: C-KIT+ (CD117)	Imatinib	Yes (20 months)	CR
MPECN	−	*P-AKT* + (IQ 100%)	Sirolimus + Sorafenib	Yes (5 months)	SD

## Data Availability

The datasets used and/or analysed during the current study available from the corresponding author on reasonable request.
